# Draft genome sequences of *Listeria monocytogenes* strains from human listeriosis in Sweden harboring premature stop codons in the virulence determinant *inlA*

**DOI:** 10.1128/mra.00464-24

**Published:** 2024-10-21

**Authors:** Sangmi Lee, Phillip Brown, Wilhelm Tham, Marie-Louise Danielsson-Tham, Gloria Lopez-Valladares, Todd Ward, Driss Elhanafi, Yi Chen, Sophia Kathariou

**Affiliations:** 1 Department of Food and Nutrition, Chungbuk National University, Cheongju, Chungbuk, South Korea; 2 Department of Plant and Microbial Biology, North Carolina State University, Raleigh, North Carolina, USA; 3 School of Hospitality, Culinary Arts and Meal Science, Örebro University, Örebro, Sweden; 4 U.S. Department of Agriculture, Agricultural Research Service, Peoria, Illinois, USA; 5 Biomanufacturing Training and Education Center, North Carolina State University, Raleigh, North Carolina, USA; 6 Division of Microbiology, Center for Food Safety and Applied Nutrition, Food and Drug Administration, College Park, Maryland, USA; 7 Department of Food, Bioprocessing and Nutrition Sciences, North Carolina State University, Raleigh, North Carolina, USA; University of Maryland School of Medicine, Baltimore, Maryland, USA

**Keywords:** *Listeria monocytogenes*, *inlA*, premature stop codon, clinical, Sweden, internalin A

## Abstract

Premature stop codons in the internalin virulence determinant *inlA* are common in serotype 1/2a *Listeria monocytogenes* from food/food processing environments but rare among human clinical isolates. Here, we report the genome sequences of serotype 1/2a (STs 121 and 3258) human listeriosis isolates from Sweden harboring such mutations in *inlA*.

## ANNOUNCEMENT

*Listeria monocytogenes* causes listeriosis, with most human cases involving serotypes 1/2a, 1/2b, and 4b (1–3). The virulence factor InlA is a surface protein harboring the LPXTG wall-anchoring motif and critical for interactions with E-cadherin in human cells (4). Premature stop codons (PMSCs) upstream of the LPXTG motif abolish such interactions, attenuating virulence (4, 5). Strains harboring such PMSCs are rare among human clinical isolates (4, 6), but serotype 1/2a, 1/2b, or 1/2c strains with *inlA* PMSCs are common in foods and food-processing environments (4–7). Here, we describe the genome sequences of four serotype 1/2a human clinical strains from Sweden, 1994–2007, harboring *inlA* PMSCs, with ST121, CC121 (n=3; T491) and ST3258, CC101 (n = 1; T56), with T491 and T56 indicating the lengths of the truncated proteins (Table 1). ST121 is considered hypovirulent (8). Identical PMSCs were identified in other strains within the same SNP clusters as these strains (Table 1).

**TABLE 1:** Characteristics of the genomes from the *L. monocytogenes* strains reported in this study

Characteristics	CFSAN003451	*CFSAN061448* ^*a*^	PNUSAL000772	RL15000682	*CFSAN061473* ^*a*^	RL15000269	*CFSAN061397* ^*a*^	CFSAN108733	CFSAN110155	*CFSAN061398* ^*a*^	CFSAN108741
Isolation year, country	NA^*b*^	2002, Sweden	NA, Germany	2011, Europe	2007, Sweden	2010, Europe	1994, Sweden	1999, Sweden	2003, Sweden	1995, Sweden	1995, Sweden
Source	NA	Human (blood)	Food (pie)	Food	Human (blood)	Food	Human (blood)	Human (blood)	Human (blood)	Human	Human (cerebrospinal fluid)
ST, CC	ST121, CC121	ST121, CC121	ST121, CC121	ST121, CC121	ST121, CC121	ST121, CC121	ST121, CC121	ST121, CC121	ST121, CC121	ST3258, CC101	ST3258, CC101
CT	ND^*c*^	CT1011	ND	ND	CT909	ND	CT14745	CT14745^*b*^	CT14745	CT14750	CT14750
SNP cluster	PDS000024645.207	PDS000024645.207	PDS000024645.207	PDS000024645.207	PDS000024645.207	PDS000024645.207	PDS000070862.4	PDS000070862.4	PDS000070862.4	PDS000135862.1	PDS000135862.1
*inlA* PMSC	T491^*d*^	T491^*d*^	T491^*d*^	T491^*d*^	T491^*d*^	T491^*d*^	T491^*d*^	T491^*d*^	T491^*d*^	T56^*e*^	T56^*e*^
Alternative strain ID		4421			6720		2155			2309	
GenBank accession no.	AABDGW000000000.1f	ABKMVB000000000.1	AAAKGY000000000.1	AAASPR000000000.1	ABKPJO000000000.1	AAASCE000000000.1	ABKMXM000000000.1	ABGAFK000000000.1	ABIQJD000000000.1	ABKMRV000000000.1	ABGAEI000000000.1
BioProject accession no.	PRJNA229903	PRJNA215355	PRJNA212117	PRJNA475189	PRJNA215355	PRJNA475189	PRJNA215355	PRJNA215355	PRJNA215355	PRJNA215355	PRJNA215355
BioSample accession no.	SAMN03354311	SAMN33143124	SAMN02924341	SAMN09500562	SAMN33246782	SAMN09500258	SAMN33143165	SAMN28032391	SAMN31152486	SAMN33143116	SAMN28026885
Assembly accession no.	PDT000102705.3	PDT001608110.1	PDT000035528.3	PDT000475196.1	PDT001612385.1	PDT000474627.1	PDT001608139.1	PDT001297967.1	PDT001442278.1	PDT001608106.1	PDT001297896.1
Raw reads accession no.	SRR1818111	SRR23353180	SRR1520048	SRR7441178	SRR23409838	SRR7440606	SRR23353213	SRR19033127	SRR21802745	SRR23353175	SRR19028292
Sequencing method	MiSeq	MiSeq	MiSeq	HiSeq 2500	NextSeq 500	HiSeq 2500	MiSeq	MiSeq	MiSeq	MiSeq	MiSeq
Number of reads (pairs)	452,617	1,056,218	609,899	2,003,929	1,342,685	2,159,041	594,680	803,057	684,795	785,290	691,494
Total length of reads (bp)	210,971,067	400,033,857	178,513,052	397,478,577	331,192,877	426,749,752	327,673,473	384,944,786	322,083,709	430,025,449	333,365,915
Draft genome length (bp)	3,040,830	3,113,227	3,033,962	3,197,293	3,138,532	3,155,710	3,035,934	3,066,835	3,057,303	3,092,497	3,012,404
Average coverage (×)	69	128	58	124	105	135	107	125	105	139	110
Average GC content (%)	38	38	38	38	38	38	38	38	38	38	38
Number of contigs	30	40	32	51	51	41	30	32	26	18	58
N50 (bp)	373,456	482,314	312,001	243,994	319,354	327,781	312,792	236,546	509,277	543,094	99,678
Protein coding sequences	3,023	3,070	3,018	3,212	3,126	3,160	2,991	3,014	3,001	3,062	2,961
Pseudogenes	34	20	32	30	20	31	15	17	18	21	21
rRNAs	3	3	5	4	4	2	3	3	3	3	5
tRNAs	61	62	46	46	57	47	61	59	51	54	57

^*a*^ Strains sequenced in this study.

^*b*^ NA, not available.

^*c*^ ND, not determined.

^*d*^ The *inlA* PMSC was caused by C > T substitution at position 1747 in the *inlA* gene, resulting in the production of 491 aa truncated InlA.

^*e*^ The *inlA* PMSC was due to the deletion of A at position 167 in the *inlA* gene, resulting in the production of 56 aa truncated InlA.

^*f*^ Links to the relevant websites are marked in the blue font.

**Fig 1:**
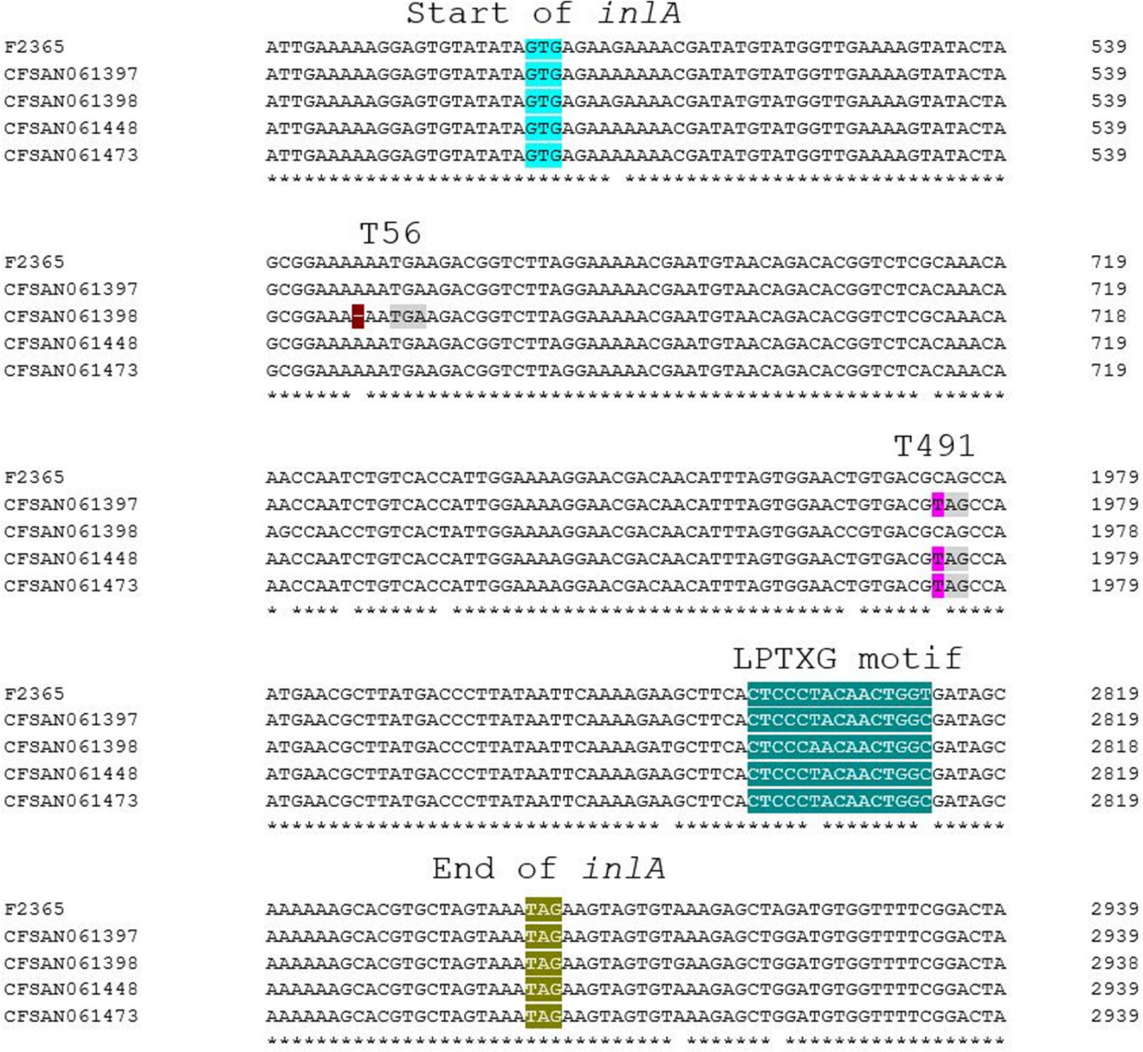
Alignment of the genomic regions from 500 bp upstream to 500 bp downstream of the *inlA* homologs in the four strains sequenced in this study. Strain F2365 is included as the reference genome. The start and stop of *inlA*, PMSCs T56 and T491, and the DNA sequence encoding the LPXTG motif are marked in light blue, green, gray, and teal, respectively. Mutations in *inlA* resulting in PMSCs T56 and T491 (deletion of A at nucleotide position 167 and a C > T substitution at nucleotide position 1747, respectively) are highlighted in brown and pink, respectively.

The strains were isolated from blood samples of the listeriosis patients by incubating the samples (10 mL) at 37°C for up to 24 h both aerobically and anaerobically and subsequently incubating 0.1 mL of samples at 37°C for 24 h on 5% bovine blood agar plates. One typical colony was examined biochemically to confirm *L. monocytogenes*, and cells were stored at −75°C in brain heart infusion (BHI) broth (Merck, Darmstadt, Germany) with 20% (vol/vol) glycerol. Genomic DNA was extracted with the DNeasy blood and tissue kit (Qiagen, Valencia, CA, USA) from overnight cultures grown at 37°C in BHI (Becton, Dickinson and Co., Sparks, MD, USA) with agitation (250 rpm). Paired-end libraries were prepared using 0.5–1 ng of genomic DNA with the Nextera XT DNA library preparation kit (Illumina, San Diego, CA, USA), and raw sequencing reads were obtained with either a NextSeq 500 sequencer with the NextSeq 500/550 high-output kit v2.5 (300 cycles, 2 × 150 bp; Illumina) or a MiSeq desktop sequencer with the MiSeq kit v2 (500 cycles, 2 × 250 bp; Illumina). Trim Sequences tools within CLC Genomics Workbench v7.5.1 (CLC bio, Boston, MA, USA) were employed to filter low-quality reads (Quality trimming), trim adaptors (Adapter trimming), and remove short reads (Sequence filtering with minimum length 50). The reads were assembled with SKESA (version 2.2) (9), and contigs were annotated with NCBI Prokaryotic Genome Annotation Pipeline (version 2021–01-11.build5132) (10). ST, CC, and cluster type (CT) designations were determined using BIGSdb-Lm (https://bigsdb.pasteur.fr/listeria) (11), and *inlA* homologs were identified via BLAST2 (version 2.12.0+) (12) against full-length *inlA* in the complete genome of *L. monocytogenes* reference strain F2365 (accession no. NC_002973.6). PMSCs were identified by comparing the length of the *inlA* homologs with F2365 *inlA* and aligning the genomic regions encompassing *inlA* homologs with Clustal Omega (version 1.2.1) (13) (Fig. 1). Default parameters were used for all software unless otherwise specified. The data suggest repeated involvement of *inlA* PMSC-harboring strains in human listeriosis in Sweden and will facilitate efforts to elucidate factors potentially compensating for truncated InlA in these strains.

## Data Availability

The genome sequences presented here are available at GenBank (Table 1).
